# Cell-free *N*-glycosylation of peptides using synthetic lipid-linked hybrid and complex *N*-glycans

**DOI:** 10.3389/fmolb.2023.1266431

**Published:** 2023-09-12

**Authors:** Lisa Wenzel, Marcus Hoffmann, Erdmann Rapp, Thomas F. T. Rexer, Udo Reichl

**Affiliations:** ^1^ Department of Bioprocess Engineering, Max Planck Institute for Dynamics of Complex Technical Systems, Magdeburg, Germany; ^2^ glyXera GmbH, Magdeburg, Germany; ^3^ Chair of Bioprocess Engineering, Otto-Von-Guericke University Magdeburg, Magdeburg, Germany

**Keywords:** multi-enzyme cascade reactions, *in vitro N*-glycosylation, synthetic glycobiotechnology, glycoengineering, oligosaccharyltransferase

## Abstract

Cell-free, chemoenzymatic platforms are emerging technologies towards generating glycoconjugates with defined and homogeneous glycoforms. Recombinant oligosaccharyltransferases can be applied to glycosylate “empty,” i.e., aglycosyalted, peptides and proteins. While bacterial oligosaccharlytransferases have been extensively investigated, only recently a recombinant eukaryotic single-subunit oligosaccharyltransferase has been successfully used to *in vitro N*-glycosylate peptides. However, its applicability towards synthesizing full-length glycoproteins and utilizing glycans beyond mannose-type glycans for the transfer have not be determined. Here, we show for the first time the synthesis of hybrid- and complex-type glycans using synthetic lipid carriers as substrates for *in vitro N*-glycosylation reactions. For this purpose, transmembrane-deleted human β-1,2 *N*-acetylglucosamintransferase I and II (MGAT1ΔTM and MGAT2ΔTM) and β-1,4-galactosyltransferase (GalTΔTM) have been expressed in *Escherichia coli* and used to extend an existing multi-enzyme cascade. Both hybrid and agalactosylated complex structures were transferred to the *N*-glycosylation consensus sequence of peptides (10 amino acids: G-S-D-A-N-Y-T-Y-T-Q) by the recombinant oligosaccharyltransferase STT3A from *Trypanosoma brucei*.

## Graphical Abstract

**Figure:**
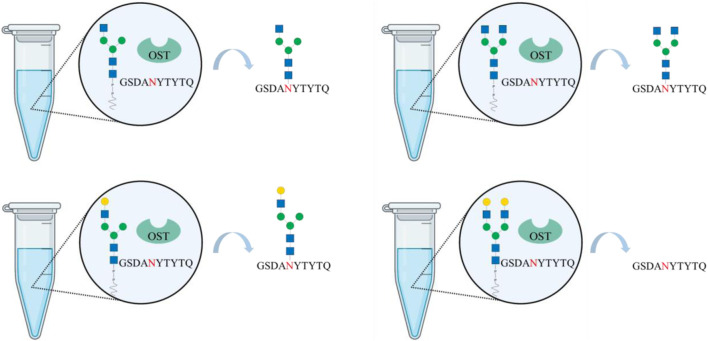
Created with BioRender.com.

## 1 Introduction

*N*-linked glycans occur in all domains of life and can affect protein stability, solubility, cell trafficking, signaling, antigenicity as well as immunogenicity (Stanley et al., 2015; Varki, 2017). However, the impact of individual glycans on function of the corresponding protein could only be elucidated poorly, so far; mainly due to both macro- and microheterogeneities (Reusch and Tejada, 2015; Schön et al., 2021). Glycoengineering methods provide options to tackle this problem as they allow the generation of defined homogeneous glycoforms. The methods can be classified into *in vivo* and *in vitro*, or cell-free approaches (Rexer et al., 2021a). *In vivo* glycoengineering refers to the genetic engineering of cells, such as knockouts, knockdowns, overexpressions and mutations. For instance, over the past decades, various efforts have been made to introduce the *N*-glycosylation machinery of eukaryotes into *Escherichia coli* or to “humanize” the glycosylation machinery of *Saccharomyces cerevisiae* and *Pichia pastoris* to produce proteins with human-like complex glycan structures for production of therapeutic proteins (Hamilton et al., 2003; Jacobs and Callewaert, 2009). Moreover, Chinese hamster ovary (CHO), and, to a minor extend, mouse myeloma (NS0) and human embryonic kidney (HEK) cell lines, have been engineered to generate homogeneous glycoforms (Heffner et al., 2021; Narimatsu et al., 2021).

An emerging alternative to cell line engineering and an option to decouple upstream processing from glycoengineering is *in vitro* glycoengineering (Warnock et al., 2005; Mastrangeli et al., 2018). This method also avoids problems related to batch-to-batch variations in glycosylation. Here, the glycans are modified after or as part of the downstream processing using recombinant enzymes, i.e., Leloir glycosyltransferases, glycosynthases or oligosaccharyltransferases (OSTs). While Leloir glycosyltransferases and glycosynthases require a glycan moiety linked to the protein, OSTs transfer glycans from lipid-linked precursors to “empty” glycosylation consensus sequences (Li and Wang, 2018).

In higher eukaryotes, OSTs are membrane associated protein complexes with two dolichyl-diphosphooligosaccharide-protein glycosyltransferase subunit (STT3) isoforms (Zufferey et al., 1995; Kelleher and Gilmore, 2005; Ruiz-Canada et al., 2009). These isoforms catalyze the transfer of lipid-linked oligosaccharides (LLOs) to the eukaryotic consensus sequence Asn-Xaa-Thr/Ser (Xaa ≠ Pro). Moreover, single-subunit OST occurs to glycosylate proteins, but only in some lower eukaryotes, prokaryotes and archaea, for instance the PglB protein in *C. jejuni* (*Campylobacter jejuni*) (Szymanski et al., 1999; Wacker et al., 2002). PglB transfers oligosaccharides to the consensus sequence Asp/Glu-Xaa1-Asn-Xaa2-Ser/Thr (with Xaa any amino acid except proline). In several studies, PglB was used to *N*-glycosylate proteins *in vitro* (Wacker et al., 2002; Glover et al., 2005). However, the amino acid sequence specificity of *C. jejuni* PglB is different to eukaryotic OSTs and its activity towards mammalian-like *N*-glycan cores for protein glycosylation is very low (<1%) (Valderrama-Rincon et al., 2012).

In the present study, the *in vitro* synthesis of novel synthetic LLOs and the transfer thereof to synthetic peptides are shown. For the former we used a set of recombinant Endoplasmic reticulum-resident glycosyltransferases [β-1,4-mannosyltransferase (ALG1ΔTM), α-1,3/1,6-mannosyltransferase (ALG2)] and a set of Golgi-resident glycosyltransferases (α-1,3-mannosyl-glycoprotein 2-β-N-acetylglucosaminyltransferase (MGAT1ΔTM), α-1,6-mannosyl-glycoprotein 2-β-N-acetylglucosaminyltransferase (MGAT2ΔTM), β-1,4-galactosyltransferase 1 (GalTΔTM), both expressed in *E. coli* or *S. cerevisiae* (Ramírez et al., 2017a; Li et al., 2018; Li et al., 2019; Rexer et al., 2020; Rexer et al., 2021b; Xiang et al., 2022). After purification by immobilized metal affinity chromatography purification (IMAC), LLOs with hybrid-type and complex-type glycans were synthesized. Finally, we investigated which of these structures could be transferred to the eukaryotic *N*-glycosylation consensus sequence Asn-Xaa-Thr/Ser (Xaa ≠ Pro) to a peptide comprising 10 amino acids, by recombinant *Trypanosoma brucei* STT3A (Ramírez et al., 2017b; Rexer et al., 2020).

To our knowledge, this is the first time, the direct transfer of hybrid and complex-type *N-*glycans to peptides has been demonstrated.

## 2 Materials and methods

### 2.1 Materials

A detailed list of all materials and chemicals used in this study can be found in Supporting Material.

### 2.2 Enzymes

All enzymes used in the study are detailed in Table 1. Except for ALG2, transmembrane deleted variants were overexpressed. Information regarding the *in vivo* and *in vitro* activity of the enzymes has been described previously (Stanley et al., 2015; Ruhnau et al., 2021).

**TABLE 1:** Enzymes used in this study and their origin; the last column states the expressed amino acids.

Name	EC number	Origin	Amino acids
ALG1ΔTM	2.4.1.142	*S. cerevisiae*	35–449
ALG2	2.4.1.257	*S. cerevisiae*	Full length (1–463)
MGAT1ΔTM	2.4.1.101	*H. sapiens*	30–445
MGAT2ΔTM	2.4.1.143	*H. sapiens*	30–447
GalTΔTM	2.4.1.38	*H. sapiens*	45–398

### 2.3 Gene expression and enzyme purification

#### 2.3.1 Gene expression of ALG1ΔTM, MGAT1ΔTM, GalTΔTM and MGAT2ΔTM in *E. coli*

The plasmid for ALG1ΔTM was transferred into and expressed by *Escherichia coli* BL21 (DE3) according to Rexer et al. (2020) (Rexer et al., 2018).

Gene sequences of human MGAT1ΔTM and GalTΔTM were inserted into the expression vector pET-28a (+); the gene sequence of MGAT2ΔTM was inserted into the expression vector pET-28b (+). Both vectors harbor an N-terminal 6-fold histidine tag (His_6_-tag) for purification. Vectors were transformed in Shuffle^®^ T7 Express *lysY* (MGAT2ΔTM) and BL21 (DE3) (MGAT1ΔTM, GalTΔTM) *E. coli* cells, respectively. Positive transformants, selected by antibiotics, were used for protein expression. In general, cultures were cultivated in terrific broth medium under agitation at 30°C. Overnight cultures were inoculated to an optical density at 600 nm (OD_600_) of 0.1. Gene expression was induced at OD_600_ = 0.6 by the addition of 0.4 mM isopropyl β-D-1-thiogalactopyranoside (IPTG) and cultures were cooled to 16°C. Cells were harvested after overnight incubation by centrifugation (7,192 x *g*, 20 min, 4°C) and cell pellets were stored at −20°C until further usage.

#### 2.3.2 Gene expression of ALG2 in *Saccharomyces cerevisiae* and membrane fraction preparation

ALG2 expression was conducted according to Rexer et al. (2020) using the yeast strain Yeast ORF ALG2 (YGL065C), which was purchased from the Dharmacon™ Yeast ORF Collection (Cambridge, United Kingdom) (Rexer et al., 2020). For gene expression, 200 mL synthetic drop-out medium without uracil was inoculated with an overnight culture to an OD_600_ = 0.3. Cultivation was performed at 30°C under agitation at 120 rpm until OD_600_ reached 1.2. Gene expression was induced by adding 100 mL yeast peptone medium supplemented with 2% galactose (final concentration). Cells were harvested after 23 h of cultivation (120 rpm, 30°C) by centrifugation (6,000 x *g*, 20 min), washed once with ice cold water and cell pellets were stored at −20°C. 3 g wet biomass were resuspended in 30 mL buffer A (30 mM Tris (pH 7.5), 3 mM MgCl_2_, 0.1% octylphenoxy poly (ethyleneoxy) ethanol (IGEPAL CA-630) and lysed at 1,000 bar for three cycles. Cell debris was removed by centrifugation for 20 min at 8,000 x *g* (4°C), followed by ultracentrifugation (100,000 x *g* for 45 min at 4°C). Membrane fractions were solubilized in 13.5 mL buffer A with 50% (v/v) glycerol and used for all synthesis reactions. This is chromatographic purification of Alg2 did not yield pure, active enzyme solutions (Rexer et al., 2020).

#### 2.3.3 Gene expression of *Trypanosoma brucei* STT3A in Sf9 cells

STT3A expression and purification were performed according to Ramírez et al. (2017) using flashback DNA (Oxford Expression Systems) and Sf9 insect cells (Merck, Darmstadt, Germany) (Ramírez et al., 2017b). A synthetic gene of *T. brucei* coding for STT3A with a 10-fold histidine and yellow fluorescent protein tag was purchased from Thermo Fisher Scientific.

#### 2.3.4 Enzyme purification

All enzymes with the exception of Alg2 were purified. For purification, cells were lysed by high-pressure homogenization (three cycles, 1,000 bar, 4°C) followed by centrifugation (7,192 x *g*, 20 min, 4°C). Supernatants were then applied to an equilibrated immobilized metal affinity chromatography (IMAC) column (Ni Sepharose HP column; Cytiva, Chicago, United States). Purified enzyme solutions were desalted using Amicon^®^ Ultra 0.5 mL filters from Merck (Darmstadt, Germany) with a molecular weight cut-off of 10 kDa according to the manufacturer’s instruction. Desalted enzymes were stored in 50% (v/v) glycerol at −20°C.

### 2.4 Synthesis of lipid-linked oligosaccharides

In general, all reactions were performed in a volume of 100 μL, at 30°C and under agitation at 300 rpm (Thermomixer comfort; Eppendorf, Hamburg, Germany). Typically, 10 µL aliquots were taken from reaction batches for analysis by multiplexed capillary gel electrophoresis with laser-induced fluorescence detection (xCGE-LIF) analysis. Glycan nomenclature is detailed in Table 2.

**TABLE 2:** All detected and referenced glycan and LLO structures, respectively. The monosaccharide buildings blocks are mannose (green circle), GlcNAc (blue square) and galactose (yellow circle). *N*-glycans are depicted according to the SNFG-nomenclature (Xiang et al., 2022).

Glycan	Structure
Man2	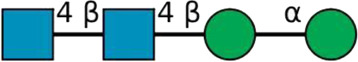
Man3	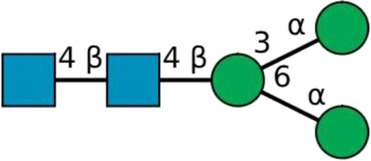
Man4	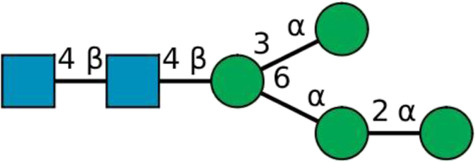
Man5	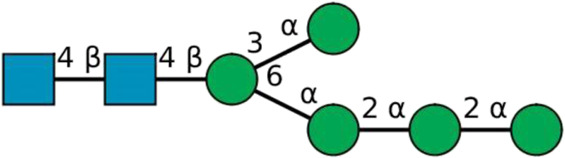
Man3GlcNAc1[3]	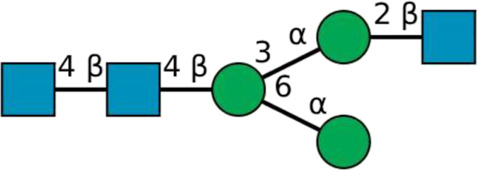
Man3GlcNAc1Gal1[3]	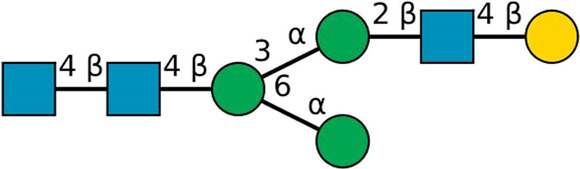
Man3GlcNAc2	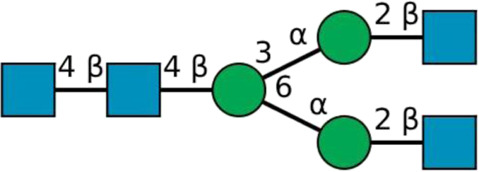
Man3GlcNAc2Gal2	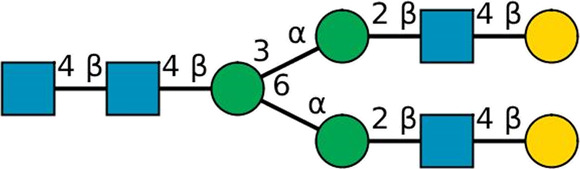

#### 2.4.1 Synthesis of LL-Man3

This one-pot multi-enzyme run was carried out for 8 h. The reaction batch contained: 50 mM 3-(Morpholin-4-yl)propane-1-sulfonic acid (pH 6.8), 0.1% IGEPAL CA-630, 10 mM MgCl_2_ (buffer B), 1 mM dithiothreitol, 0.1 mM phytanyl-PP-chitobiose, 2 mM GDP-mannose, 0.1 mg/mL purified ALG1ΔTM and 35% (v/v) ALG2 yeast membrane fraction. After 8 h, the reaction batch was quenched for 5 min at 90°C to ensure enzyme inactivity.

#### 2.4.2 Synthesis of hybrid structures: LL-Man3GlcNac1[3] and LL-Man3GlcNAc1Gal1[3]

The first reaction contained 25 mM HEPES (pH 7), 0.1% IGEPAL and 10 mM MnCl_2_ (buffer C) with 4 mM UDP-N-acetylglucosamine (UDP-GlcNAc), 50% (v/v) LL-Man3 and 1 mg/mL MGAT1ΔTM. After 24 h, reactions were quenched for 5 min at 90°C. The second reaction, to synthesize LL-Man3GlcNAcGal1[3] was conducted in buffer C, 4 mM UDP-galactose, 50% (v/v) LL-Man3GlcNAc1[3] and 0.84 mg/mL GalTΔTM. The reaction was performed for 24 h and was quenched at 90°C for 5 min.

#### 2.4.3 Synthesis of complex structures: LL-Man3GlcNac2 and LL-Man3GlcNac2Gal2

*Sequential synthesis:* The first reaction batch contained buffer C with 4 mM UDP-GlcNAc, 50% (v/v) Man3 and 1 mg/mL MGAT1ΔTM and MGAT2ΔTM, respectively. After 24 h, reactions were quenched at 90°C for 5 min and centrifuged. The second reaction batch contained: buffer C, 4 mM UDP-galactose, 0.84 mg/mL GalTΔTM and 50% (v/v) LL-Man3GlcNac2. The reaction was quenched at 90°C for 5 min after 24 h of incubation.

*One-pot synthesis*: The one-pot synthesis of LL-Man3GlcNac2Gal2 was performed in buffer C with 4 mM UDP-GlcNAc, 4 mM UDP-Gal, 35% (v/v) LL-Man3 and 1 mg/mL MGAT1ΔTM, MGAT2ΔTM and GalTΔTM, respectively. The reaction was performed for 36 h at 30°C under agitation (350 rpm). After 0, 4, 8, 24 and 36 h, aliquots of 10 µL were taken and the reaction was quenched for 5 min at 90°C.

### 2.5 Transfer of LLOs to synthetic peptides by *Trypanosoma brucei* STT3A

To analyze the transfer of LLOs 5-carboxytetramethylrhodamine (TAMRA)-labeled synthetic peptides with the consensus sequence Asn–Xaa–Thr/Ser and the following amino acid sequence, G-S-D-A-**N-Y-T**-Y-T-Q, were purchased from Biomatik (Cambrigde, Canada). The reaction was conducted in a volume of 100 µL and contained: 20 mM HEPES (pH 7.5), 10 mM MnCl_2_, 150 mM NaCl, 0.035% (w/v) DDM, 0.007% (w/v) CHS, 50% (v/v) LLO, 20 µM synthetic peptides, 1.8 mg/mL *T brucei* STT3A and EDTA-free protease inhibitor (Roche, Basel, Switzerland).

### 2.6 Glycan analysis

#### 2.6.1 Multiplexed capillary gel electrophoresis with laser induced fluorescence detection

The synthesis of LLOs was analyzed by xCGE-LIF (Schwarzer et al., 2008; Ruhaak et al., 2010; Hennig et al., 2016; Cajic et al., 2021). Glycans were released from the lipid tail by mild acidic hydrolysis using 50 mM HCl for 30 min at 90°C and neutralized with 100 mM NaOH. Afterwards freeze-dried glycans were labeled with 8-aminopyrene-1,3,6-trisulfonic acid (APTS) and excess APTS was removed by hydrophilic interaction chromatography with solid phase extraction (HILIC-SPE). For analysis, 1 µL sample, 9.6 µL HiDi™, 0.7 µL LIZ™ base pair standard and 0.7 µL glyXalign™ GA2 (glyXera GmbH, Magdeburg, Germany) for orthogonal migration time normalization were mixed, electrokinetically injected and separated after transfer to a glyXboxCE™-system (glyXera; modified 3130 Genetic Analyzer, Life Technologies, California, United States) equipped with a POP7™ polymer matrix filled 4-capillary array (50 cm). Data analysis was carried out using glyXtoolCE™ (glyXera).

#### 2.6.2 Mass spectrometry

Prior to the analysis by mass spectrometry, enzymes and larger molecules were separated from the reaction products by filtration using a 10 kDa molecular weight cut-off filters (Amicon^®^ Ultra 0.5 mL filters; Merck, Darmstadt, Germany). Samples were desalted by manual C18 solid-phase extraction. Therefore, samples were reconstituted in up to 1 mL 0.1% trifluoroacetic acid (TFA) to ensure that pH is lower than 3. HyperSEP C18 columns (ThermoFisher Scientific) were conditioned with 3 mL of conditioning buffer (90% methanol, 10% H_2_O, 0.1% TFA). Prior to sample loading the column was equilibrated with 0.1% TFA in water. Samples were desalted using 0.1% TFA in water and glycopeptides were eluted with 50% acetonitrile in water containing 0.1% TFA. For detergent removal HiPPR™ spin columns (ThermoFisher Scientific) were used according to the manufacturer’s instruction. Sample measurement and glycoproteomic analysis were conducted as described previously (Rexer et al., 2020). Briefly, TAMRA glycopeptides were analyzed by reverse-phase liquid chromatography coupled online to RP-LC-ESI-OT-OT MS/MS (LTQ Orbitrap Elite hybrid mass spectrometer, Thermo Fisher Scientific) using higher-energy collision dissociation fragmentation (HCD) at a normalized collision energy of 20 (NCE 20, HCD. low). Glycopeptide mass spectra were evaluated using Xcalibur Qual Browser 2.2 (Thermo Fisher Scientific) and Byonic search engine (Protein Metrics, San Carlos, CA). Relative quantification of TAMRA glycopeptides was performed using Byologic^®^ software (Protein Metrics).

## 3 Results

In the following, we demonstrate the cell-free synthesis of hybrid and complex-type LLOs. Furthermore, we show that recombinant *T. brucei* OST can be used to transfer *N*-glycan moieties from the lipid anchor to a peptide.

### 3.1 Synthesis of LL-Man3

**FIGURE 1:**
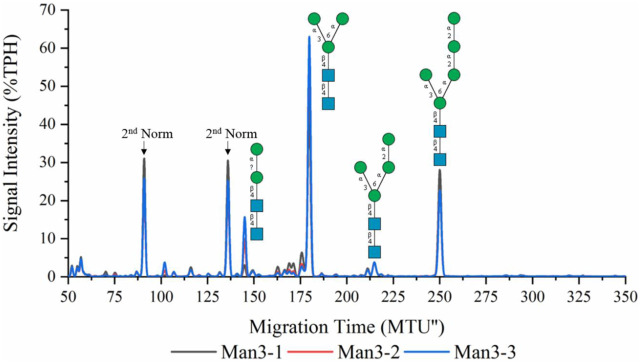
xCGE-LIF generated *N*-glycan fingerprint (=normalized/aligned electropherogram) of the LL-Man3 synthesis. The reaction was conducted in triplicates (Man3-1/2/3). Internal LIZ™ and glyXalign™ GA2 standards were used for orthogonal migration time normalization/alignment, data processing and analysis (glycan structure identification and assignment) was performed using glyXtoolCE. *N*-glycans are depicted according to the SNFG-nomenclature (Neelamegham et al., 2019).

Recombinant ALG1ΔTM and ALG2 were used for the synthesis of LL-Man3 from phytanyl-PP-chitobiose. Results of the xCGE-LIF measurement after a reaction time of 8 h are depicted in Figure 1. The electropherogram shows a large fraction of the product [65% total peak height (TPH)], LL-Man3 (178 MTU”), but also small amounts of LL-Man2 (15% TPH) at 140 MTU” and LL-Man5 (25% TPH) at 248 MTU”. The latter is most likely formed due to the naturally occurring ALG11 in the yeast membrane fraction of recombinant ALG2 (Rexer et al., 2020).

### 3.2 Synthesis of hybrid structures: LL-Man3GlcNAc1[3] and LL-Man3GlcNAc1Gal1[3]

**FIGURE 2:**
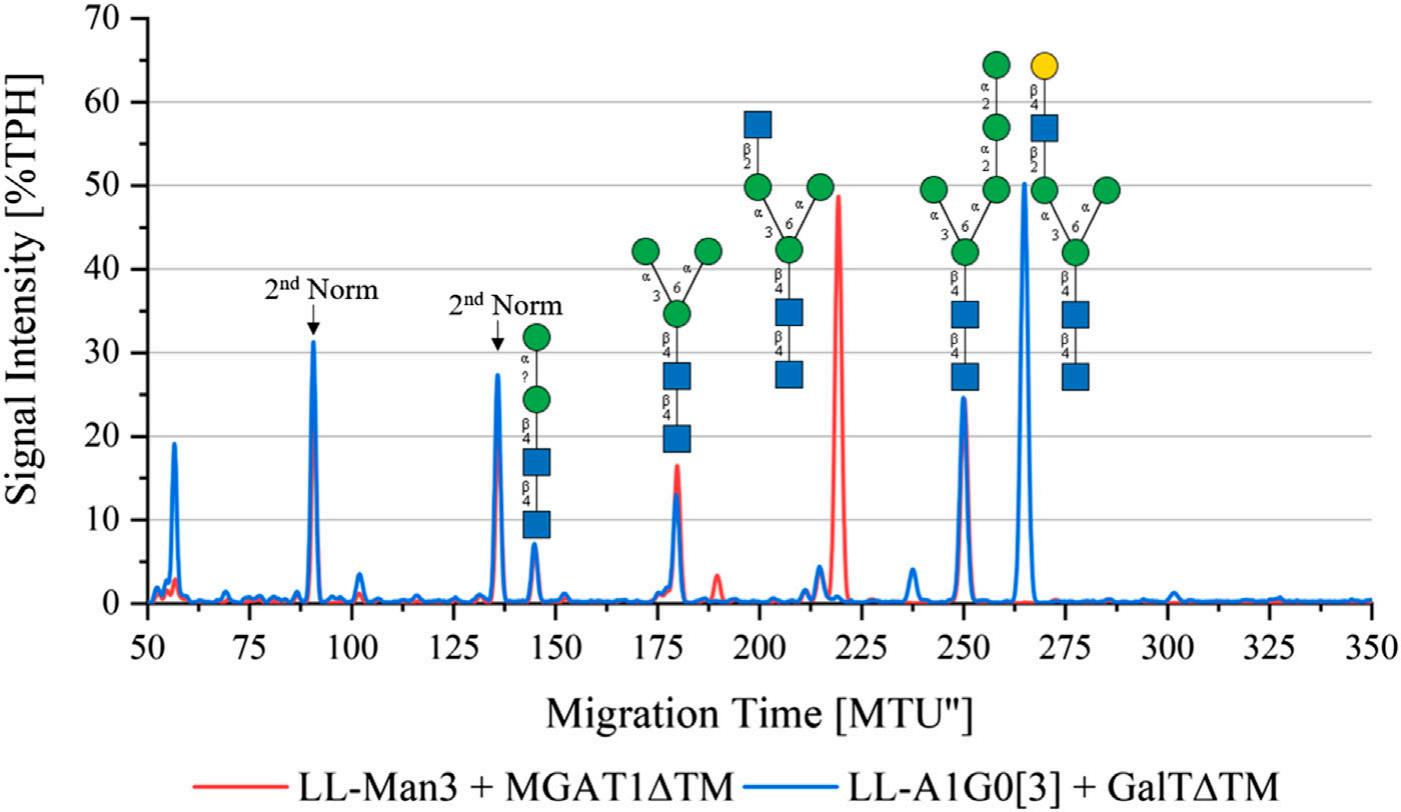
xCGE-LIF generated *N*-glycan fingerprint (=normalized/aligned electropherogram) showing the synthesis of LL-Man3GlcNAc1[3] with MGAT1 (red) and LL-Man3GlcNAc1Gal1[3] with MGAT1 + GalTΔTM (blue).

To synthesize LL-Man3GlcNAc1[3], the substrates UDP-GlcNAc and LL-Man3 plus purified MGAT1ΔTM were incubated for 24 h. Figure 2 (red) shows that after 24 h a large proportion (50%) of LL-Man3 was converted to LL-Man3GlcNAc1[3] (217 MTU”). It is unclear whether a reaction equilibrium was obtained or whether the reaction was still ongoing. However, the result is in line with previous experiments by our group (Ruhnau et al., 2021). Moreover, small amounts of intermediates such as LL-Man2 (7% TPH) (140 MTU”) and LL-Man5 (25% TPH) (248 MTU’’) that originate from the LL-Man3 stock solution were identified.

To synthesize LL-Man3GlcNAc1Gal1[3], the reaction product from the previous reaction, LL-Man3GlcNAc1[3], was incubated with UDP-galactose and GalTΔTM. Results of the reaction after 24 h are shown in blue in Figure 2. LL-Man3GlcNAc1[3] from the previous reaction (red) was fully converted to LL-Man3GlcNAc1Gal1[3] and appeared at approximately 260 MTU” in the electropherogram.

### 3.3 Synthesis of complex structures: LL-Man3GlcNAc2 and LL-Man3GlcNAc2Gal2

**FIGURE 3:**
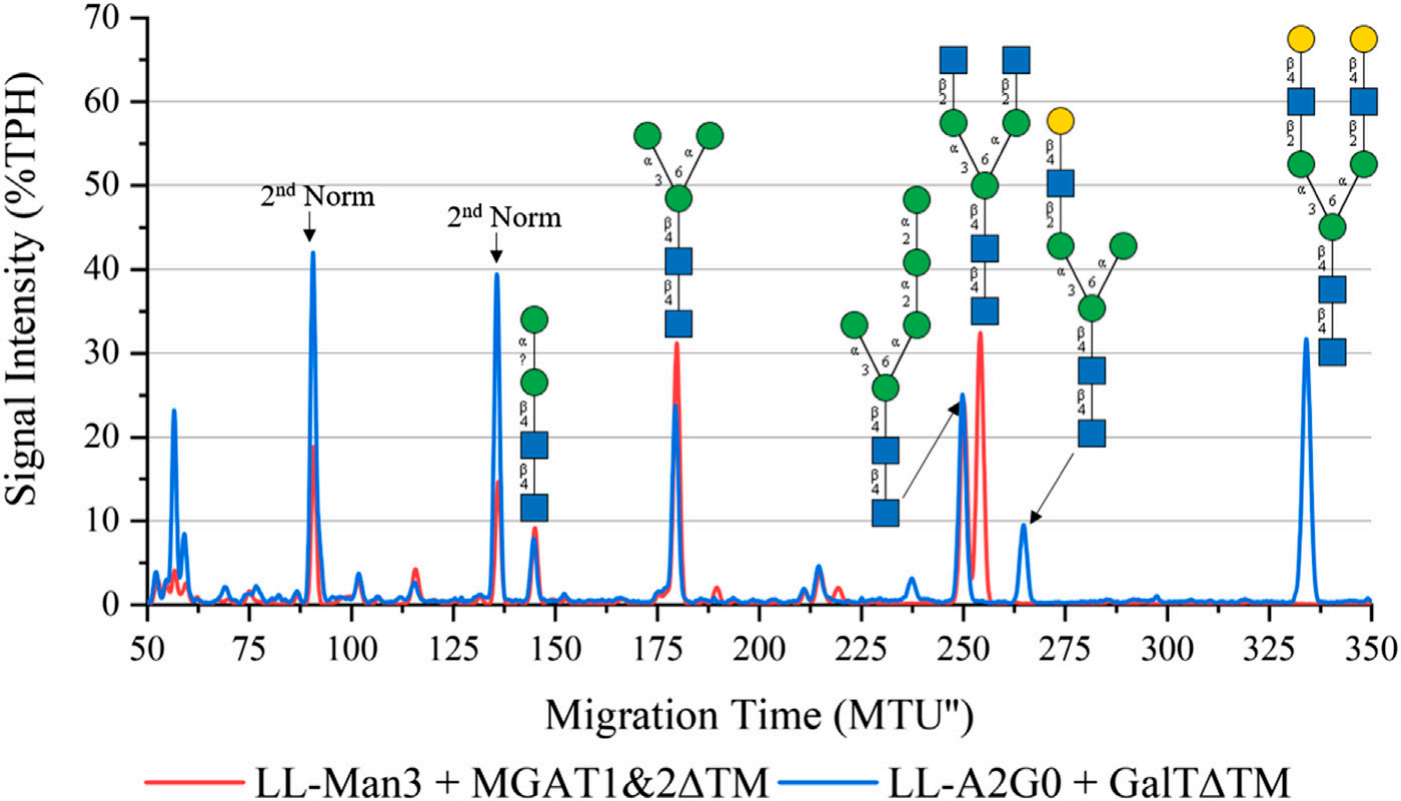
xCGE-LIF generated *N*-glycan fingerprint (=normalized/aligned electropherogram) showing the synthesis of LL-Man3GlcNAc2 with MGAT1+2 (red) and LL-Man3GlcNAc2Gal2 with MGAT1+2 and GalTΔTM (blue).

To synthesize the complex-type structure LL-Man3GlcNAc2, the enzymes MGAT1ΔTM and MGAT2ΔTM were employed in a one-pot batch reaction for 24 h (Figure 3, red). Around 50% TPH of LL-Man3 was converted to LL-Man3GlcNac2 (252 MTU”). The fact that the intermediate product LL-Man3GlcNAc1[3] (218 MTU”) was not detected implies that the conversion by MGAT2∆TM was instant. The side product LL-Man3GlcNAc1[6], a single GlcNAc residue attached to the α1-6-mannosylated antenna by MGAT2∆TM, was also not detected suggesting that there is no or only a minor activity of MGAT2∆TM towards LL-Man3.

To synthesize LL-Man3GlcNAc2Gal2, the reaction product of the previous reaction (LL-Man3GlcNAc2) was subjected to GalTΔTM and UDP-galactose. As depicted in Figure 3 (blue), LL-Man3GlcNAc2 was fully converted to LL-Man3G1cNAc2Gal2 (331 MTU’‘) Moreover, LL-Man3GlcNAc1Gal1[3] (262 MTU’’) was also present even though the substrate LL-Man3GlcNAc1[3] was not detected in the previous reaction. Hence, we suspect that the MGAT1∆TM-catalyzed reaction was still proceeding after addition of GalTΔTM and that the galactosylation occurs faster than the attachment of GlcNAc by MGAT2∆TM. Moreover, it appears that the activity of MGAT2∆TM towards LL-Man3GlcNAc1Gal1[3] is either very low or absent.

**FIGURE 4:**
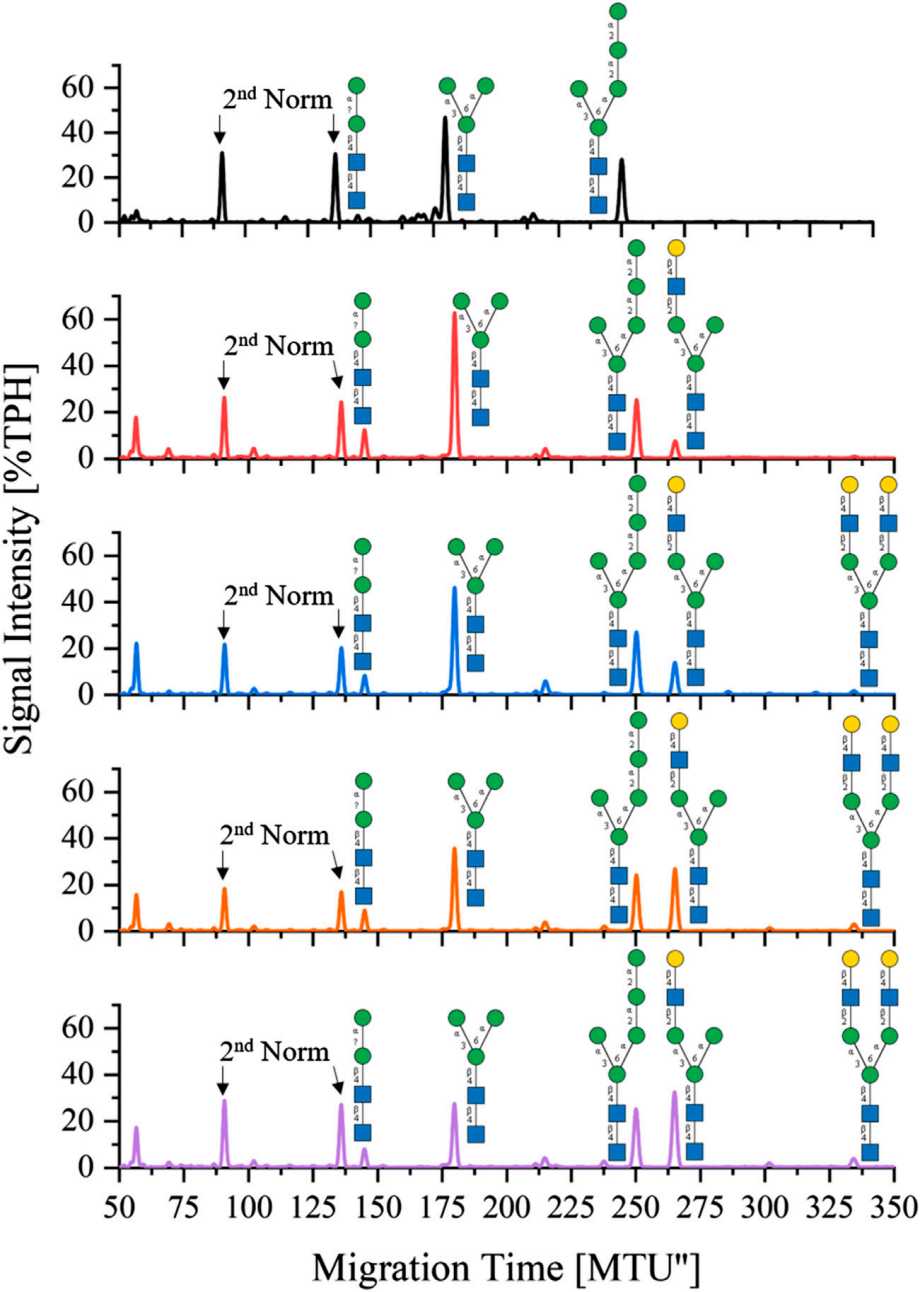
xCGE-LIF generated N-glycan fingerprints (=normalized/aligned electropherograms) showing the one-pot synthesis of LL-Man3GlcNAc2Gal2 at 0 h (black), 4 h (red), 8 h (blue), 24 h (orange) and 36 h (violet).

*One-pot synthesis of LL-Man3GlcNAc2Gal2*: To reduce the complexity of the synthesis, the production of LL-Man3GlcNAc2Gal2 was also tested in a one-pot reaction. For this purpose all enzymes (MGAT1ΔTM, MGAT2ΔTM and GalTΔTM) were added at the start of the reaction. Samples were analyzed by xCGE-LIF after 4 h, 8 h, 24 h and 36 h reaction time. Results of the reaction are depicted in Figure 4. After 4 h, LL-Man3 was partly converted to LL-Man3GlcNAc1Gal1[3]. Within the next 4 h, the reaction was driven towards the side product LL-Man3GlcNAc1Gal1[3] (262 MTU”) and the final product LL-Man3GlcNAc2Gal2 [3/6] (331 MTU”). The peak intensity of LL-Man3GlcNAc2Gal2 after 24 h is slightly higher than after 8 h. After 36 h, the reaction showed no differences in comparison to the 24 h sample. At the end of the experiment, the reaction mix mainly contained LL-Man3GlcNAc1Gal1[3] (262 MTU’’). Only a small amount of the target structure LL-Man3GlcNAc2Gal2 was obtained (∼5% TPH).

### 3.4 *In vitro* glycosylation of synthetic peptides by *Trypanosoma brucei* STT3A

Four one-pot *in vitro* glycosylation runs were conducted, to investigate the ability of recombinant *T. brucei* STT3A to transfer the synthesized hybrid (LL-Man3GlcNAc1[3], LL-Man3GlcNAc1Gal1[3]) and complex-type structures (LL-Man3GlcNac2, LL-Man3GlcNac2Gal2 [3/6]), from the phytanyl anchor to the asparagine residue of the *N*-glycosylation consensus motif of the selected peptides (Section 2.3.4). Transfer reactions were performed using the unpurified LLOs from the sequential synthesis reactions (see Figure 2, Figures 3). The results were analyzed by liquid chromatography coupled mass spectrometry (LC-MS/MS).

For all reactions, the amount of not *N*-glycosylated or deamidated peptides was high with approximately 90%. Therefore, in the following, only glycosylated peptides were considered to determine the relative proportion of transferred glycans (Table 1). Around 64% of all glycosylated peptides contained a Man5 *N*-glycan structure. LL-Man5 was present in equal amounts in all reaction batches (see Figure 2, Figures 3). As LL-Man5 was only present in small amounts, the large proportion of glycosylated Man5-peptides suggests a high affinity of *T. brucei* OST towards LL-Man5.

**FIGURE 5:**
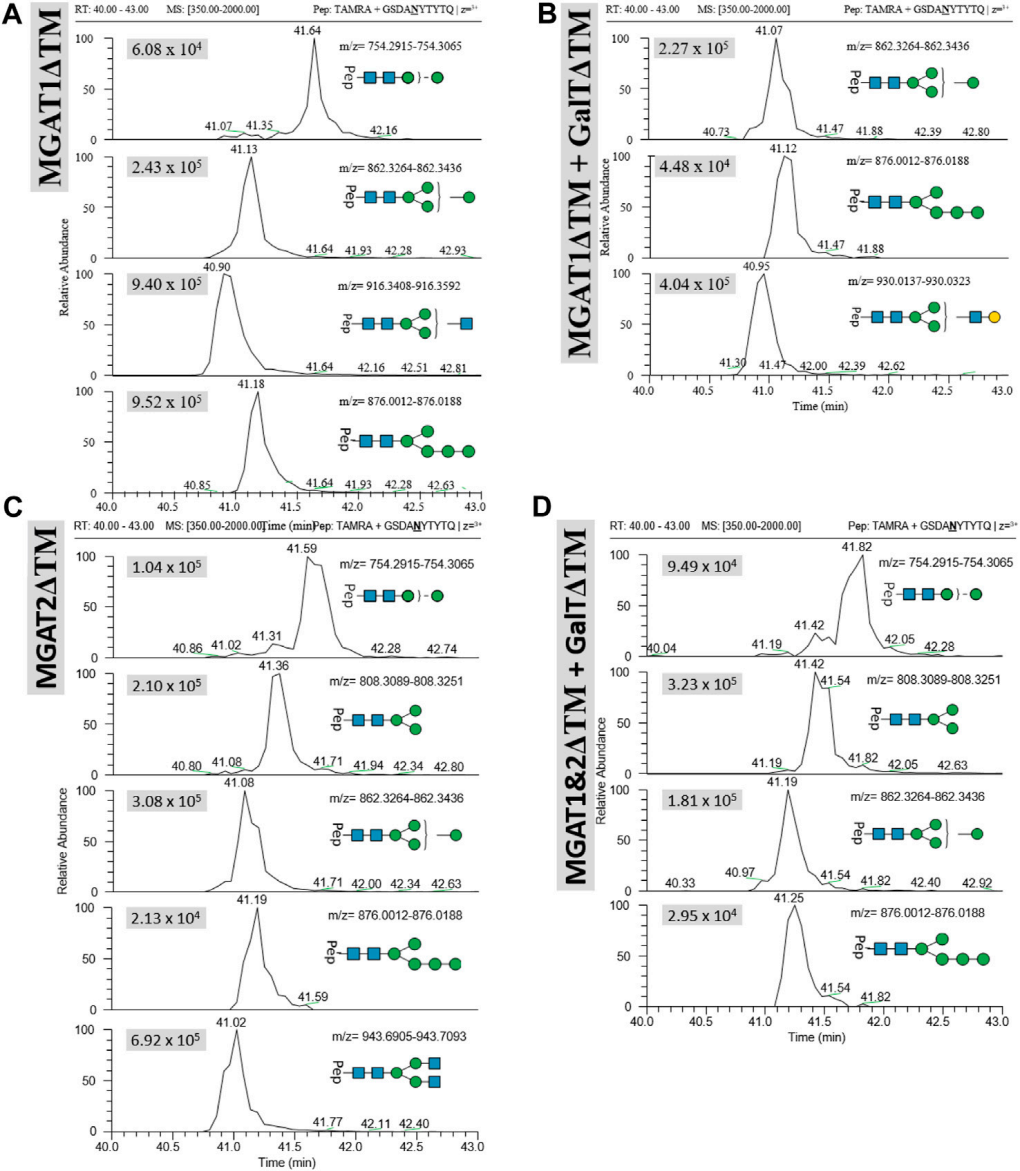
Extracted ion chromatograms (EIC) of LC-MS/MS-based *N*-glycoproteomic analysis for relative quantification of *in vitro* *N*-glycosylated synthetic TAMRA peptides for four different reactions using the following LLOs as main substrates **(A)**: Man3GlcNAc1[3], **(B)** LL-Man3GlcNAc1Gal1[3], **(C)** Man3GlcNAc2 and **(D)** Man3GlcNAc2Gal2. Extracted ion chromatograms of the precursor ions (EIC MS^1^) of TAMRA + GSDANYTYTQ + *N*-glycan are depicted for each enzymatic reaction and show the retention and relative abundance of the different *N*-glycoforms. For each EIC the peak intensity is given in arbitrary units (e.g., 9.25 × 10^5^) for the respective glycopeptide precursor ions. Only EIC-MS^1^ precursor ions with MS^2^ spectra are selected.

*Transfer of LL-Man3GlcNAc1[3]*: The extracted ion chromatograms (EIC-MS^1^) of identified glycopeptide spectra after the *in vitro* glycosylation reaction are depicted in Figure 5A. Peptides with Man2, Man4, Man5 and LL-Man3GlcNAc1[3] *N*-glycan structures were identified. The fraction of glycopeptides containing LL-Man3GlcNAc1[3] was around 30% (see Table 3).

**TABLE 3:** LC-MS/MS-based *N*-glycoproteomic analysis of the *in vitro* *N*-glycosylation of synthetic and TAMRA-labeled glycopeptides via *T. brucei* OST. The composition and structures of the detected N-glycans present on the TAMRA-labeled peptide are displayed in the first two columns of the table. The relative proportion of TAMRA glycopeptides in % is displayed on the right; [-] product not detectable in the reaction batch. For each reaction all detected N-glycopeptide are listed with their relative proportion (only glycosylated peptides were considered for the calculation).

Modification		TAMRA glycopeptide [relative proportion in %]			
		MGAT1ΔTM	MGAT1ΔTM + GalTΔTM	MGAT1+2ΔTM	MGAT1+2ΔTM + GalTΔTM
HexNAc(2)Hex (2)	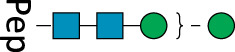	1.4	0.0	3.1	5.0
HexNAc(2)Hex (3)	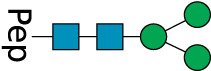	0.0	0.0	5.4	24.2
HexNAc(2)Hex (4)	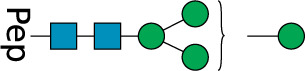	7.4	9.4	12.4	7.1
HexNAc(3)Hex (3)	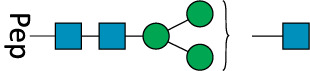	30.0	-	–	–
HexNAc(2)Hex (5)	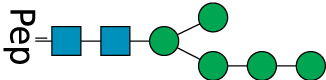	61.1	69.6	60.1	63.7
HexNAc(3)Hex (4)	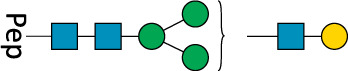	–	21.1	–	0.0
HexNAc(4)Hex (3)	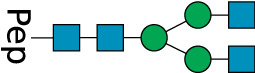	–	–	18.8	–

*Transfer of LL-Man3GlcNAc1Gal1[3]*: Peptides with Man4, Man5 and Man3GlcNAc1Gal1[3] *N*-glycan structures were identified by LC-MS/MS measurement (Figure 5B). The glycosylation reaction yielded the LL-Man3GlcNAc1Gal1[3]-peptide with a proportion of around 21% (see Table 3).

*Transfer of Man3GlcNAc2*: The complex-type Man3GlcNAc2 could successfully be transferred by STT3A to the synthetic peptide (see Figure 5C; Table 1). As in the reaction described before, side-products were identified. The relative proportion of the Man3GlcNAc2peptide was around 19% of all glycosylated peptides in this reaction.

*Transfer of Man3GlcNAc2Gal*2*:* This fully galactosylated structure could not be detected on any peptide after the glycosylation reaction by STT3A implying that the activity of STT3A towards this complex structure is very low or absent (Figure 5D; Table 3).

## 4 Discussion

To investigate key properties of therapeutic peptides and proteins, methods to generate homogeneous and defined glycoforms efficiently would be very helpful (Jefferis et al., 2021; Schön et al., 2021). *In vitro* (chemo-) enzymatic methods are especially useful as they do not require complex modifications of host cells or time consuming bioreactor cultivations but can be applied independently using a range of bacterial and mammalian Leloir glycosyltransferases that are already available (Li and Wang, 2018; Chao et al., 2020; Jaroentomeechai et al., 2020; Kightlinger et al., 2020; Rexer et al., 2021c; Jaroentomeechai et al., 2022). In addition, glycosidases can be engineered to allow for specific transglycosylation reactions (Wang et al., 2019). However, among all (chemo-) enzymatic approaches, only recombinant OSTs can transfer glycans to “empty,” i.e., aglycosylated consensus sequences by reconstituting and reducing *in vivo N*-glycosylation pathways *in vitro*. While bacterial OSTs like *C*. *jejuni* PglB have been extensively investigated, it has only recently been shown that an active recombinant eukaryotic single-subunit OST can be obtained using an insect cell expression system (Ramírez et al., 2017b). Hence, it is now possible to *in vitro N*-glycosylate the eukaryotic consensus sequence consensus Asn-Xaa-Thr/Ser (Xaa ≠ Pro) of peptides. As substrates for the glycosylation reaction, lipid-linked glycans are required and a range of saturated and unsaturated synthetic lipid-linked carriers have been synthesized and successfully used to replace natural lipid carriers that are not soluble in water. Thus, the complexity of the *in vitro* reaction can be reduced significantly (Ramírez et al., 2017a; Boilevin and Reymond, 2018; Rexer et al., 2018; Li et al., 2019; Rexer et al., 2020; Xiang et al., 2022). However, while *in vivo* eukaryotic OSTs transfer (high-)mannose-type glycans, their use for transfer of hybrid- and complex-type *N*-glycans *in vitro* has not been investigated. Here, for the first time, complex *N*-glycan moieties were synthesized on synthetic lipid carriers. In particular, four novel LLOs–LL-Man3GlcNAc1[3] LL-Man3GlcNAc1Gal1[3], LL-Man3GlcNAc2 and LL-Man3GlcNAc2Gal2—were synthesized. Analysis of the reaction batches demonstrated that all final products were present as the main component in the reaction mix. In the future, these new LLOs and derivatives, for example, sialylated structures, can be used to investigate a range of novel OSTs that are either obtained through protein engineering or enzyme mining for their potential to transfer hybrid and complex-type *N*-glycan structures (Napiórkowska et al., 2018). However, while all enzymes could readily be expressed in *Escherichia coli*, the availability and costs of activated sugars and lipid-linked chitobiose are additional barriers for the synthesis of these LLOs at preparative scale. In particular, lipid-linked chitobiose is not commercially available and can only be obtained through elaborate chemical synthesis (Boilevin and Reymond, 2018).

*In vitro N-*glycosylation reactions using recombinant *T. brucei* SST3A demonstrated that the structures Man3GlcNAc1[3] and Man3GlcNAc1Gal1[3] as well as the complex structure GlcNAc2Man3GlcNAc2 can be transferred to synthetic peptides. In comparison to other studies, however, the fraction of glycosylated over aglycosylated peptides after the reaction was very low (Ramírez et al., 2017b). As previously pointed out, this could be caused by the use of a fully saturated lipid carrier and possibly be alleviated by using unsaturated lipids as carriers (Liu et al., 2014; Rexer et al., 2020). As expected, STT3A had a significantly higher affinity to LL-Man5 than for hybrid and complex LLOs. As, the cascade reactions for LLO assembly could not be controlled to the extent to yield individual structures only, developing a method for LLO separation will be required to provide defined, pure LLOs for use as substrates for *in vitro* glycosylation reactions. Additionally, purified human Alg2 expressed in HEK cells lines can be used as alternative to avoid LL-Man5 formation by residual Alg11 (Ramírez et al., 2017a). Furthermore, the potential of OSTs, in particular STT3A, to *in vitro* glycosylate longer and more complex peptides, or even proteins, should be determined in future studies.

Altogether, the presented results offer a new synthetic approach for the *in vitro* glycosylation of aglycosylated peptides at the eukaryotic *N*-glycosylation consensus sequence with hybrid and complex structures using chemoenzymatically preassembled LLOs.

## Data Availability

The raw data supporting the conclusion of this article will be made available by the authors, without undue reservation.
