# Supplementary material for: Cell-free N-glycosylation of peptides using synthetic lipid-linked hybrid and complex N-glycans
Source: Front Mol Biosci. 2023 Sep 12;10:1266431. doi: 10.3389/fmolb.2023.1266431 (PMC10520871; doi:10.3389/fmolb.2023.1266431)
Supplement: Supplementary file 1 [file DataSheet1.DOCX]

**Supplementary Information**

Table S 1: List of abbreviations

| % (v/v) | volume percent |
| --- | --- |
| ACN | Acetonitrile |
| ALG | Asparagine-linked glycosylation |
| ALG1 | β-1,4-mannosyltransferase |
| ALG1ΔTM | Transmembrane deleted β-1,4-mannosyltransferase |
| ALG2 | α-1,3/1,6-mannosyltransferase |
| APTS | (3-Aminopropyl)triethoxysilane |
| GalTΔTM | β-1,4-galactosyltransferase 1 |
| CGE-LIF | capillary gel electrophoresis with laser-induced fluorescence detection |
| CHS | 3β-Hydroxy-5-cholestene 3-hemisuccinate |
| DDM | n-Dodecyl-β-D-maltopyranoside |
| DTT | Dithiothreitol |
| *E. coli* | *Escherichia coli* |
| ER | Endoplasmic reticulum |
| Gal | Galactose |
| GDP | Guanosine diphosphate |
| GlcNAc | N-Acetylglucosamine |
| GT | Glycosyltransferase |
| HiDi | Hi-Di™ Formamide |
| HILIC | Hydrophilic interaction chromatography |
| IMAC | Immobilized metal affinity chromatography |
| IPTG | Isopropyl β-D-1-thiogalactopyranoside |
| LC-MS | Liquid Chromatography coupled mass spectrometry |
| LLO | Lipid-linked oligosaccharide |
| Man | Mannose |
| MGAT1ΔTM | α-1,3-mannosyl-glycoprotein 2-β-N-acetylglucosaminyltransferase |
| MGAT2ΔTM | α-1,6-mannosyl-glycoprotein 2-β-N-acetylglucosaminyltransferase |
| MgCl2 | Magnesium chloride |
| MnCl2 | Manganese chloride |
| MWCO | Molecular weight cut off |
| OD600 | Optical density at 600 nm |
| OST | Oligosaccharyltransferase |
| PTM | Post-translational modification |
| rpm | Rounds per minute |
| *S. cerevisiae* | *Saccharomyces cerevisiae* |
| ssOST | Single-subunit oligosaccharyltransferase |
| TAMRA | 5-Carboxytetramethylrhodamine |
| TFA | Trifluoroacetic acid |
| UDP-Gal | Uridindiphosphate-galactose |
| UDP-GlcNAc | Uridindiphosphate-N-Acetylglucosamine |
| YFP | Yellow fluorescent protein |

Table S 2: List of materials and companies

| **Chemical** | **Company** |
| --- | --- |
| 2nd NormMix | glyXera GmbH |
| GalTΔTM in pET-28a(+) | BioCat |
| Acetonitril | VWR Chemicals |
| Agar-Agar powder | Carl Roth GmbH |
| APTS | Sigma-Aldrich |
| Baculovirus Insect Cell Medium | Novagen r |
| BioGel® P-10 Media | Bio-Rad |
| BL21(DE3) Competent *E.coli* | New England Biolabs GmbH |
| CHS | Sigma-Aldrich |
| DDM | Thermo Fisher ScientificTM |
| DTT | Sigma-Aldrich |
| Ethanol | Carl Roth GmbH |
| GDP-Man | Sigma-Aldrich |
| GeneScanTM 500 LIZ® Size Standard | Applied Biosystems® |
| Glycerol | Carl Roth GmbH |
| HCl | VWR Chemicals |
| HEPES | Carl Roth GmbH |
| Hi-DiTM Formamide | Thermo Fisher ScientificTM |
| IGEPAL® CA-630 | Sigma-Aldrich |
| Imidazol | Sigma-Aldrich |
| IPTG | Sigma-Aldrich |
| Isopropanol | Sigma-Aldrich |
| K2HPO4 | Carl Roth GmbH |
| Kanamycin sulfate | Carl Roth GmbH |
| KH2PO4 | Carl Roth GmbH |
| Lemo21(DE3) Competent *E.coli* | New England Biolabs GmbH |
| L-rhamnose | New England Biolabs GmbH |
| Magnesium chloride | Sigma-Aldrich |
| Manganese chloride | Merck |
| Methanol | Fisher Scientific |
| MGAT1ΔTM in pET-28a(+) | BioCat |
| MGAT2ΔTM in pET-28b(+) | BioCat |
| MOPS | Carl Roth GmbH |
| NaOH | Carl Roth GmbH |
| Phytanyl-PP-Chitobiose | Chiroblock |
| Pierce BCA Protein Assay Kit | Thermo Fisher ScientificTM |
| Reducing Agent | glyXera GmBH |
| *S. cerevisiae* ORF ALG2 (YGL065C) | Horizon |
| Shuffle T7 Express LysY Competent *E. coli* | New England Biolabs GmbH |
| Sodium chloride | Carl Roth GmbH |
| TEA | AppliChem Panreac |
| TFA | Sigma-Aldrich |
| Tryptone | Carl Roth GmbH |
| UDP-Gal | Sigma-Aldrich |
| UDP-GlcNac | Sigma-Aldrich |
| Yeast extract | Carl Roth GmbH |
